# A rare association of tuberculous longitudinally extensive transverse myelitis (LETM) with brain tuberculoma

**DOI:** 10.1186/s40064-015-1232-z

**Published:** 2015-09-04

**Authors:** Rajendra Singh Jain, Sunil Kumar, Shankar Tejwani

**Affiliations:** Department of Neurology, Sawai Man Singh Medical College, Jaipur, Rajasthan India; Department of Radiology, Sawai Man Singh Medical College, Jaipur, Rajasthan India

**Keywords:** Tuberculous myelitis, Longitudinally extensive transverse myelitis (LETM), Brain tuberculoma, Tuberculosis

## Abstract

**Background:**

Longitudinally extensive transverse myelitis is characterized by contiguous inflammatory lesion of spinal cord involving three or more spinal segments. It is a well-recognized but rare presentation of Mycobacterium tuberculosis infection.

**Case description:**

We report a case of young boy diagnosed with multiple brain tuberculomas. He was on antitubercular drugs therapy for 2 months and became asymptomatic. On 2-month followup visit, the patient complained of acute onset progressive sensorimotor, spastic paraparesis with bladder dysfunction. Magnetic resonance imaging of spine showed longitudinally extensive transverse myelitis extending from thoracic spinal segment T2 to T10 level. He was treated with high dose intravenous methylprednisolone therapy and continued on combination of first line four antitubercular drugs. At 6-month followup, patient was able to walk with support. In our patient, clinical features, previous history of brain tuberculoma and spinal neuroimaging confirmed the diagnosis of tuberculous myelitis. The new onset longitudinally extensive transverse myelitis in our patient was may be related to paradoxical response to antitubercular therapy.

**Conclusions:**

Our case highlights that tubercular infection might be an important but overlooked cause of longitudinally extensive transverse myelitis. Therefore, clinicians should have a high index of suspicion to diagnose this potentially treatable cause especially in high-risk conditions like tuberculosis endemic areas, associated brain tuberculosis and HIV infection. Our case is unique because of paradoxical presentation of longitudinally extensive transverse myelitis in cranial tuberculomas, already on antitubercular treatment.

## Background

The most common central nervous system (CNS) manifestations of TB is tuberculous meningitis (95 %), followed by cerebral tuberculoma and tuberculous abscess. Other infrequent manifestations are calvarial tuberculosis, tuberculous pachymeningitis (Tariq and Ahmed 2012) and spinal arachnoiditis (Naidoo et al. 1991). Intramedullary spinal tuberculosis is an uncommon presentation. There have been few case reports of intramedullary spinal tuberculosis in literature (Lin et al. 1994). Longitudinally extensive transverse myelitis (LETM) is characterized by contiguous immune-mediated inflammatory lesion of spinal cord extending to three or more spinal cord segments (West 2013). Tuberculosis is a rare cause of LETM.

## Case description

**Fig. 1:**
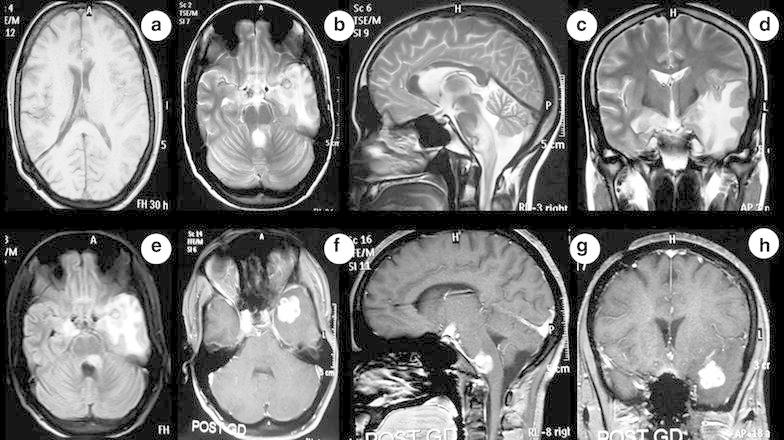
MRI of brain shows multiple intraparenchymal lesions involving brainstem and anterior temporal lobe. These lesions are isointense to grey matter on T1-weighted (**a**), hyperintense on T2-weighted (**b**, **c**, **d**) and fluid attenuated inversion recovery (FLAIR) (**e**) images. There is no central hyperintensity on T2-weighted image. Surrounding perilesional vasogenic edema is present. Gadolinium-enhanced T1-weighted images (**f**, **g**, **h**) shows multiple conglomerated ring enhancement. Ventriculoperitoneal shunt in situ is seen in right lateral ventricle

An 18-year old boy had mild grade fever, headache, recurrent vomiting and confusion for 2 months. The magnetic resonance imaging (MRI) of brain showed multiple intraparenchymal lesions involving brainstem and anterior temporal lobe. These lesions were isointense on T1-weighted (Fig. 1a), hyperintense on T2-weighted (Fig. 1b–d) and fluid attenuated inversion recovery (FLAIR) (Fig. 1e) images. There was no central hyperintensity on T2-weighted image. Perilesional vasogenic edema was present and Gadolinium-enhanced T1-weighted images (Fig. 1f–h) showed multiple ring enhancing conglomerate lesions. He was diagnosed as multiple intracranial tuberculomas with hydrocephalus. Ventriculoperitoneal shunt surgery was done and patient was started on first line antitubercular drugs (isoniazid, rifampin, pyrazinamide and ethambutol) along with corticosteroids and pyridoxine. At 2 month follow-up visit, he complained of acute onset weakness in both lower limbs and urinary incontinence for 5 days. There was no history of headache, fever, visual blurring, seizures, altered sensorium or noncompliance to treatment. On examination, higher mental functions, speech and cranial nerves including bilateral fundi were normal. The muscle power was medical research council (MRC) grade 2/5 in both lower limbs and normal in upper limbs. Deep tendon reflexes (knee and ankle) were exaggerated (+++) and Babinski sign were positive in both lower limbs. All the modalities of sensation including pain, touch, temperature, joint position and vibration were impaired below thoracic 5 (T5) spine level. There were no meningeal or cerebellar signs.

Hemogram, biochemistry including liver functions, renal functions, thyroid function tests and serum vitamin B12 level were normal. Skin tuberculin sensitivity test was positive. Erythrocyte sedimentation rate (ESR) was raised (54 in first hour). Computed tomography (CT) of thorax and abdomen were normal. Serum ELISA test for human immunodeficiency virus (HIV) was negative. The markers for autoimmune and connective tissue disorders (ANA, Anti-ds DNA, Anti-nucleosome, Anti-histones, Anti-Sm, Anti SS-A, Anti RO, Anti Scl-70, Anti Rib-P-Protein, Anti-JO, Anti-SS-B) were negative.

**Fig. 2:**
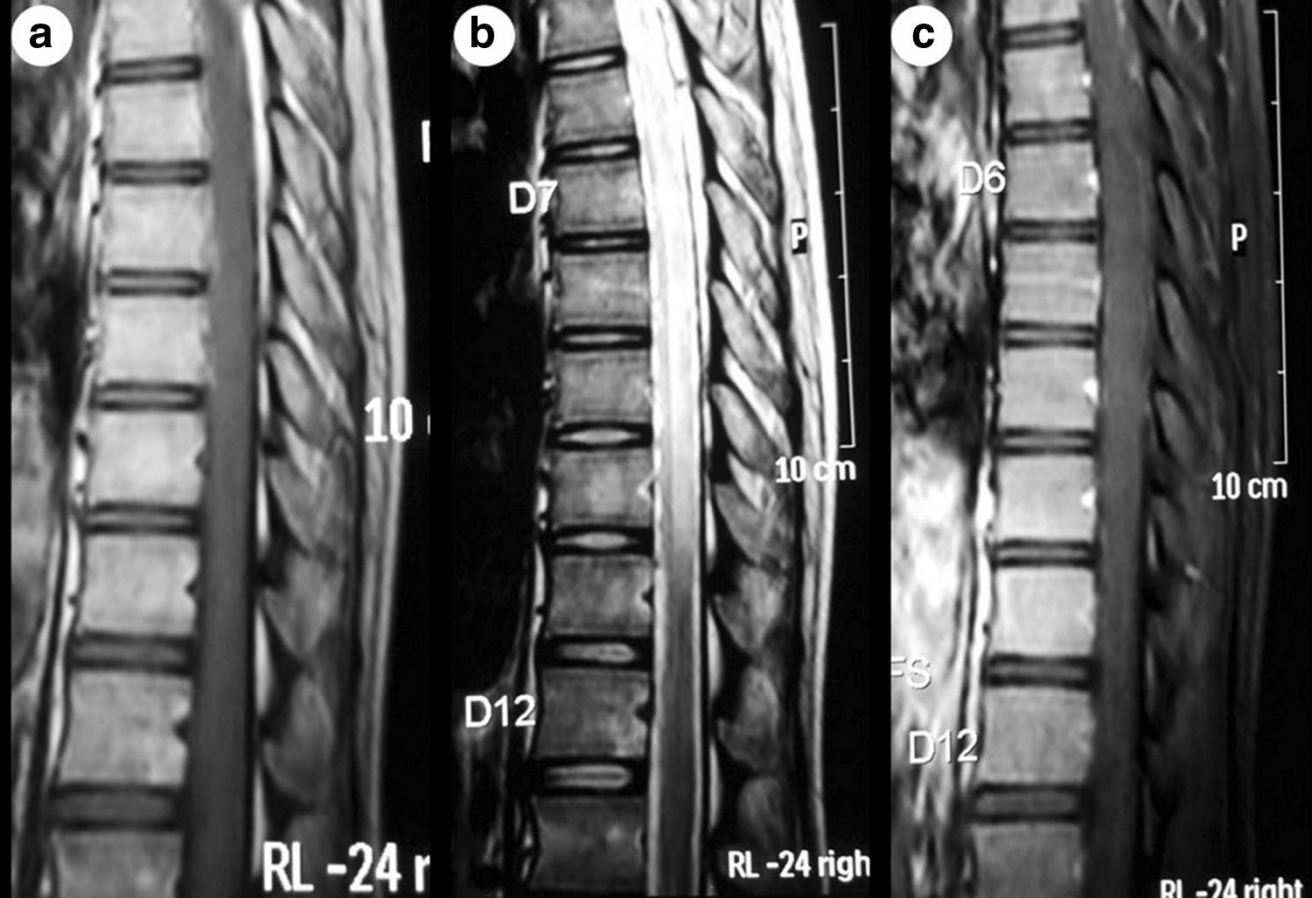
Magnetic resonance imaging (MRI) of thoracic spine (sagittal section) showing contiguous long segment intramedullary lesion which is isointense on T1-weighted (**a**) and hyperintense on T2-weighted (**b**) images extending from thoracic vertebral level T2 to T10. There is no cord expansion or contrast enhancement (**c**)

**Fig. 3:**
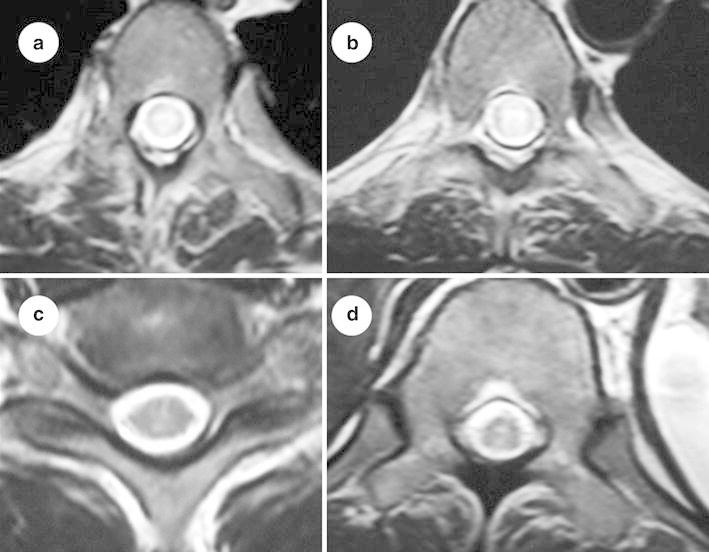
T2-weighted MRI of thoracic spine (axial section) showing poorly delineated intramedullary hyperintense lesion (**a**, **b**, **c**, **d**). There is no cord expansion

The magnetic resonance imaging (MRI) of thoracic spine showed contiguous long segment intramedullary lesion, which was isointense on T1-weighted and hyperintense on T2-weighted images extending from thoracic vertebral level T2 to T10. There was no cord expansion or contrast enhancement (Figs. 2a–c, 3a–d). The MRI features were suggestive of longitudinally extensive transverse myelitis (LETM). The MRI of brain showed no new changes.

Lumbar puncture showed normal intracranial pressure (140 mm H_2_O). Cerebrospinal fluid (CSF) analysis revealed 75 cells/mm^3^ with lymphocytic pleocytosis (Lymphocyte: 90 %, Neutrophils: 10 %), elevated protein (150 mg/dL) and normal glucose (46 mg/dL) levels. CSF acid-fast bacillus (AFB) stain was negative. Culture for Mycobacterium tuberculosis and other bacterias were negative. Polymerase chain reaction (PCR) for tuberculosis and and Herpes simplex virus were negative. CSF anti-AQP4 antibody (NMO antibodies) and oligoclonal bands were negative.

The clinical and imaging findings were consistent with the diagnosis of tuberculous LETM [T2-T10] in already existing brain tuberculomas. The patient was treated with high dose intravenous methylprednisolone (1000 mg per day for 5 days) and antitubercular drugs which patient was taking continued as such. At the time of discharge, he showed mild improvement in muscle power MRC grade 3/5 in both lower limbs. He was discharged on four antitubercular drugs on (isoniazid 300 mg, rifampicin 450 mg, pyrazinamide 1500 mg and ethambutol 800 mg), along with pyridoxine (40 mg) and dexamethasone (12 mg) per day. Dexamethasone dose was tapered off over 8 weeks. Our plan was to continue on these antitubercular drugs for 12 months and then shift to two antitubercular drug (rifampin and isoniazid) for next 6 months. At 6 months followup, he was able to walk with minimal support.

## Discussion and evaluation

Wadia and Dastur gave the term tuberculous radiculomyelitis (TBRM), which included spinal cord complications of TBM, arachnoiditis, spinal tuberculoma or granuloma (Wadia and Dastur 1969; Wadia 1973). There are few case reports describing the radiological features of tuberculous myelopathy (Gupta et al. 1994). These lesions are mostly hypointense to isointense on T1-weighted and hyperintense on T2-weighted images without contrast enhancement. The largest series of intramedullary spinal tuberculosis (15 patients) showed varied MRI changes (Ramdurg et al. 2009). Cerebrospinal fluid (CSF) analysis in tuberculous myelitis usually shows an active inflammatory response with predominantly lymphocytic pleocytosis and high protein level (Wadia and Dastur 1969).

In our patient clinical features, previous history of brain tuberculoma and spinal neuroimaging confirmed the diagnosis of tuberculous myelitis. However, other differential diagnosis like infective (bacterial, viral, parasitic), spinal cord tuberculoma, demyelinating disorders (MS, NMO), autoimmune and connective tissue disorders, vascular, neoplastic or paraneoplastic etiologies were also kept in consideration (Kitley et al. 2012). Spinal neuroimaging findings ruled out possibilities of tuberculoma, tumors or vascular lesions. Various markers for autoimmune and connective tissue disorders like ANA, Anti-ds DNA, Anti-nucleosome, Anti-histones, Anti-Sm, Anti SS-A, Anti RO, Anti Scl-70, Anti Rib-P-Protein, Anti-JO, Anti-SS-B were found to be negative in our patient. Culture for Mycobacterium tuberculosis and other bacterias were negative. Polymerase chain reaction (PCR) for Herpes simplex virus were negative. Anti-AQP4 antibody and oligoclonal bands, which are specific for neuromyelitis optica (NMO) and multiple sclerosis, respectively were negative in our patient (Trebst et al. 2011).

The patient showed marked clinical improvement of brain tuberculoma on a combination therapy of four antitubercular drugs and dexamethasone for 2 months. Though, radiological resolution of tuberculomas occur usually after 1 year of antituberculous therapy (Awada et al. 1998). Our patient developed tuberculous LETM despite good compliance to therapy. The spinal lesion was new in our patient as MRI of spine was completely normal 2 months back. It may be related to paradoxical response of antitubercular treatment. The paradoxical response during antitubercular drug therapy is characterized by clinical worsening or appearance of new lesions on imaging in a patient who had initially shown response and clinical improvement improved with the therapy (Cheng et al. 2002). The etiopathogenesis of this paradoxical response is still not clear, but various hypotheses have been proposed (Cheng et al. 2003; Rao et al. 1995). According to one hypothesis, antitubercular drug therapy causes lysis and release of tubercular protein, which mediate an intense inflammatory reaction and result in new or expansion of the preexisting lesions (Gamage et al. 2000). Other theory suggest an immune reaction to tubercular bacilli or a primary tuberculous infection. Another possibility is that the antitubercular drug have poor penetration into the CSF once the initial inflammation subsides. Another possible explanation may be non-effectiveness of antituberculous drugs due to poor CSF penetration once the initial inflammation subsides. The most likely mechanism of LETM in our patient was related to inflammatory reaction to degraded tuberculous proteins due to chemotherapy against Mycobacterium tuberculosis bacilli, which causes appearance of new lesions.

A prompt recognition and treatment is necessary to prevent irreversible damage and morbidity. There is a definite indication of corticosteroids in treatment of brain tuberculoma (Wadia and Dastur 1969). Four first line anti-tuberculosis drugs (isoniazid, rifampin, pyrazinamide and ethambutol) along with pyridoxine for prolonged period (18 months) are recommended for brain tuberculoma (van der Harst and Luijckx 2011; Gropper et al. 1995). However, no clear cut guidelines are available for management of tuberculous myelitis. Since it is believed to be an inflammatory reaction, high dose intravenous methylprednisolone might have a beneficial role in tuberculous LETM. Corticosteroids are therefore useful to treat both tuberculous LETM and brain tuberculoma in conjunction with continued use of antituberculous drugs.

## Conclusion

Longitudinally extensive transverse myelitis (LETM) is characterized by contiguous inflammatory lesion of spinal cord involving three or more spinal cord segments. LETM is a well-recognized but rare presentation of Mycobacterium tuberculosis infection. However, paradoxical tuberculous LETM in association with brain tuberculomas is unique presentation. Our case highlights that tuberculosis might be an important but neglected cause of myelitis. Therefore, clinicians should have a high index of suspicion to diagnose the potentially treatable tuberculous myelitis especially in high-risk conditions like tuberculosis endemic area, associated brain tuberculosis (meningitis, abscess and tuberculoma), HIV infection and also watch out for paradoxical response to antitubercular drug therapy.

## Consent

The patient and his parents gave written consent for the use of personal and medical information for the publication of this case report and any accompanying images.

## Abbreviations

MRI: magnetic resonance imaging
CT: computed tomography
LETM: Longitudinally extensive transverse myelitis
CSF: cerebrospinal fluid
